# A Giant Lymphatic Cyst of the Transverse Colon Mesentery

**DOI:** 10.21699/ajcr.v1i1.7

**Published:** 2010-08-14

**Authors:** Tayyaba Batool, Soofia Ahmed, Jamshed Akhtar

**Affiliations:** Department of Pediatric Surgery, National Institute of Child Health Karachi, Pakistan

**Keywords:** Lymphatic cyst, Mesenteric cyst, Lymphatic malformation, Abdominal mass

## Abstract

Mesenteric cysts are not uncommon in pediatric age group but giant lymphatic cysts of mesentery are reported infrequently. This is a report of six years old female who had vague abdominal pain with distension for two years. Investigations revealed a large cystic mass in abdomen. On exploration a giant lymphatic cyst in the mesentery of transverse colon found. More than 1500 ml of milky fluid was drained. The cyst was unilocular and appeared to be the collection of lymph (chyle) between two leaves of the mesentery of transverse colon. It is postulated that trauma to or malformation of lymphatics at the root of mesentery might have lead to this pathology.

## INTRODUCTION

Mesenteric cyst is a term applied to any cyst found in mesentery. Lymphatic cyst is a type of mesenteric cyst which is of lymphatic origin. It is usually a benign lesion. A closely related pathology is cystic hygroma which is also of lymphatic origin. This is usually found in the retroperitoneum. These are more common in small bowel mesentery but rarely reported in colonic mesentery [1 , 2].

Lymphatic cysts usually contain pale yellow fluid. Chylous cysts are infrequently seen. Chylous cysts are classified as rare variants of mesenteric cysts. It constitutes 7.3% to 9.5% of all abdominal cysts [2].

We report a case of giant mesenteric cyst at unusual location and with unique features.

## CASE REPORT

A six years old female child weighing 12 kg, admitted with history of gradual abdominal distension that was noticed by the mother for the last 2 years but did not seek any medical advice as there were no other associated complaints. With an increase of abdominal girth and vague abdominal pain, parents took the child to a nearby general practitioner who advised ultrasound and referred the child to our facility. On examination child appeared comfortable with abdominal fullness. Abdominal examination did not reveal a distinct mass though vague fullness was present oriented more in vertical direction. There was a definite feel of fluid thrill on palpation.

Ultrasound done previously showed cystic mass in abdomen. With female gender a differential diagnosis included ovarian cyst in addition to mesenteric and omental cyst. CT scan abdomen was advised. This showed a large cystic mass occupying almost whole of the abdomen and located under anterior abdominal wall with viscera pushed posteriorly (Fig. 1,2). A diagnosis of omental cyst was made at this stage.

**Figure:**
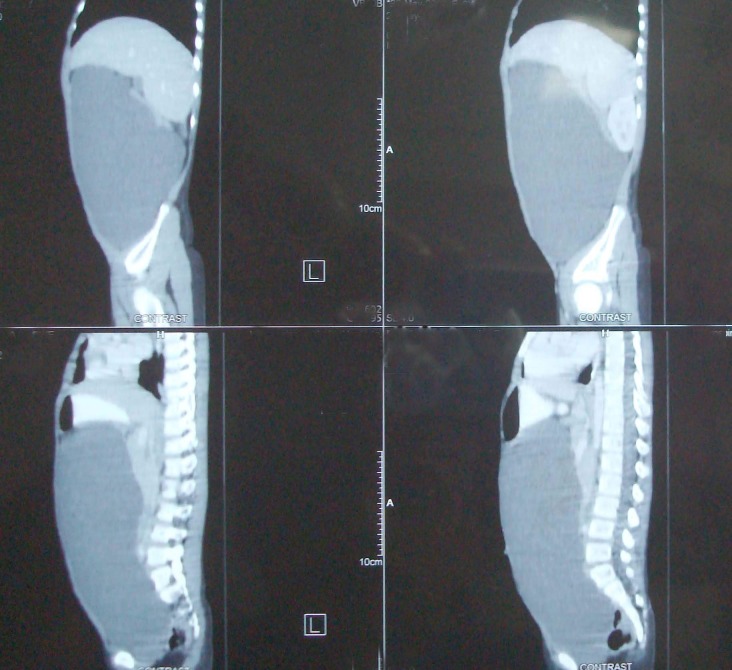
Figure 1: CT scan, saggital section showing huge unilocular cystic abdominal mass.

**Figure:**
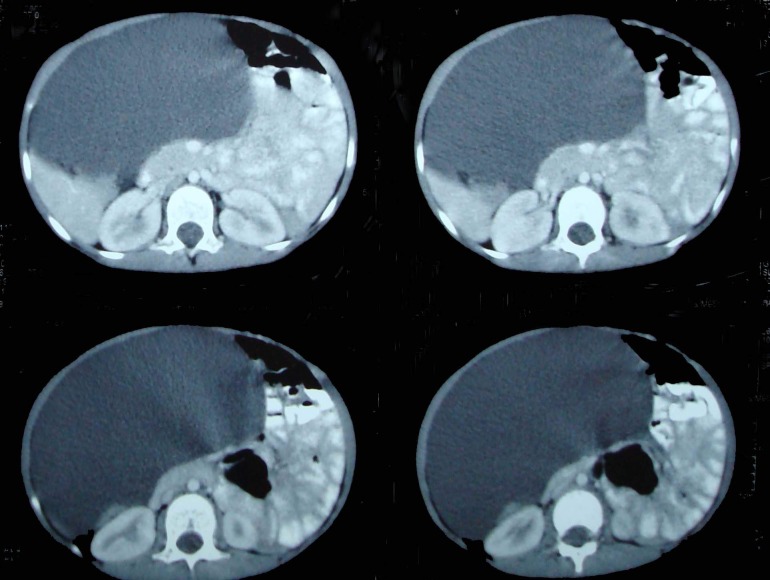
Figure 2: CT scan showing position of the cyst just beneath the anterior abdominal wall with compression of the adjacent viscera.

Laparotomy was performed. On opening peritoneum a huge lymphatic cyst was found (Fig. 3). It was delivered out with difficulty and that too after partially evacuating it. More than 1500 ml of milky fluid was drained out. The anatomy was then identified more clearly. It was found between leaves of transverse colon mesentery and extending into its root (Fig. 4). The redundant leaves of mesentery were excised without jeopardizing blood supply of colon and left open so as to prevent re-accumulation of fluid. Drain was kept and wound closed.

**Figure:**
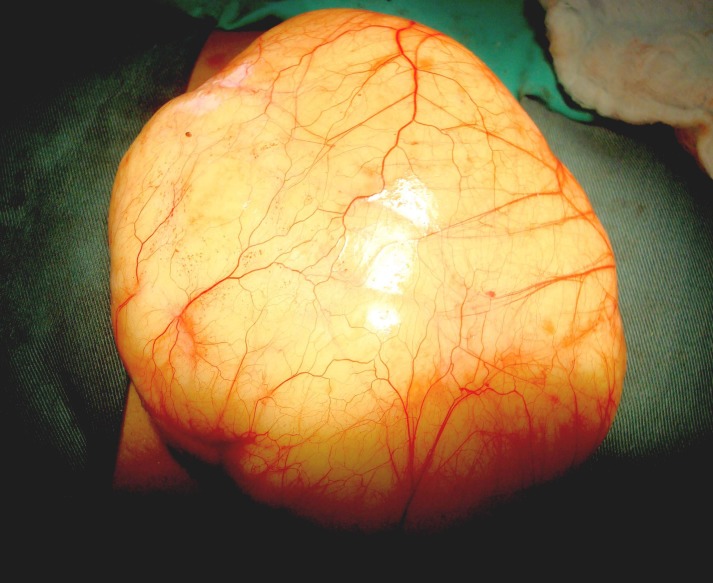
Figure 3: Operative view of chylous cyst.

**Figure:**
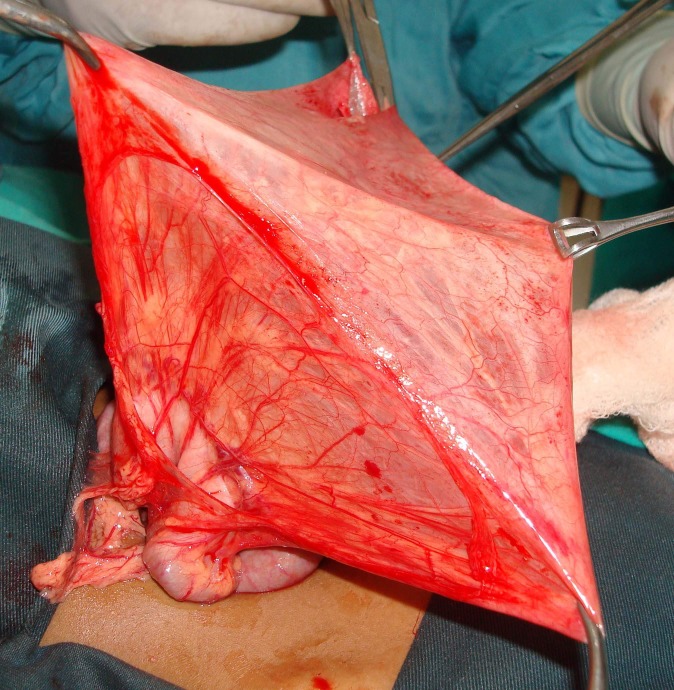
Figure 4: Stretched out leaves of mesentery following drainage of fluid.

Post-operative course was uneventful and patient discharged on 4th day. Biopsy was reported as consistent with mesenteric cyst. Parents were counseled again as to possible recurrence of the lesion and a regular follow up was planned. At three months follow up child is well. On ultrasound a small hypoechoic area of 4cm x 4cm was noted in mid abdomen. A repeat ultrasound after a month is advised to note the progress of the lesion.

## DISCUSSION

Cyst of lymphatic origin can be found within peritoneum (omentum, mesentery) as well as in the retroperitoneal area. One of the theories proposed that these cysts represent benign proliferations of ectopic lymphatics. These channels lack communication with the main lymphatic system. Other theory suggested that due to the failure of joining venous system embryonic lymph channels gradually dilate. Still one of the theories suggested that as a result of non-fusion of leaves of mesentery, lymphatic fluid accumulates within this, supposedly a dead space. They can also result from trauma to the lymphatic channels [3]. Usually these cysts are multilocular or multiseptated. Huge unilocular cysts are rare. In the patient reported here it was a huge unilocular cyst within the leaves of the mesentery of transverse colon.

These cysts mostly remain asymptomatic and only come into attention when they cause abdominal fullness / distension or vague discomfort. In this patient it caused fullness and mild abdominal pain because of which parents seek advice. Though chronic symptoms predominate acute presentation is not uncommon which includes intestinal obstruction, volvulus, hemorrhage into cyst, rupture etc. [4 , 5]. Clinical examination at times may not pick these cysts as distinct masses, rather a feeling of ascites (fluid accumulation) may be the only sign, as happened in our patient. 

Abdominal ultrasonography usually provides working diagnosis in these patients and other investigations may be avoided though a more detailed picture can be provided by CT scan abdomen which may help in planning surgery. In reported patient CT scan revealed excellent details of the lesion. Thus where possible this modality may be used for diagnostic purposes.

v Surgery is usually a straight forward affair. At times lesion can be removed completely without sacrificing any adjacent organ though resection of involved segment of small bowel, if limited area is involved, is more appropriate. The lesions of the mesentery can be excised as complete as possible, though in difficult cases and if cysts are at critical location de-roofing of the cysts is another approach, a type of marsupialization. In our patient the leaves of mesentery were extremely stretched out. Redundant part was excised easily. No well defined cyst found in this case. The histopathology reported the walls of lesion as of mesenteric origin with mesothelial lining. Considering lack of well defined limits of the lesion there are chances of recurrence of cysts in such cases. A regular follow up is thus advised. In our patient same is being followed. 

This case had many unusual features, namely being unilocular, of huge size containing milky fluid, in the mesentery of transverse colon and with apparently no distinct cystic structure. It appeared that mesenteric leaves simply stretched out due to accumulation of fluid. Presence of milky fluid within transverse colon mesentery may be explained on the basis of either malformed ruptured lymphatic channels of some trauma that might have caused leak of lymphatic fluid at the base of mesentery. This case may add to host of postulations related to lymphatic mesenteric cysts.

## Footnotes

**Source of Support:** Nil

**Conflict of Interest:** None declared
